# Intraoperative hypotension trajectories and their predictive value for major postoperative complications: a retrospective cohort study

**DOI:** 10.3389/fmed.2025.1739832

**Published:** 2026-01-14

**Authors:** Yingbo Ren, Chang Liu, Xin Wang, Mengwei Zhang, Hongyan Li

**Affiliations:** Department of Anesthesiology, The Affiliated Traditional Chinese Medicine Hospital, Southwest Medical University, Luzhou, China

**Keywords:** intraoperative hypotension, blood pressure trajectory, group-based trajectory modeling, postoperative complications, perioperative risk prediction

## Abstract

**Background:**

Intraoperative hypotension (IOH) is a common hemodynamic disturbance during major non-cardiac surgery, yet the prognostic significance of different temporal blood pressure patterns remains unclear. This study aimed to identify distinct IOH trajectories using group-based trajectory modeling (GBTM) and to evaluate their independent and incremental predictive value for major postoperative complications in high-risk surgical patients.

**Methods:**

We conducted a retrospective cohort study of 789 adults undergoing elective major abdominal, urologic, or gynecologic surgery between January 2018 and December 2023. Continuous invasive minute-by-minute mean arterial pressure (MAP) recordings were extracted from the anesthesia information management system. IOH was defined as MAP <65 mmHg. GBTM modeled MAP over absolute intraoperative time using polynomial time functions to identify three latent IOH trajectories based on duration and recurrence: transient mild (<10 min), moderate sustained (10–30 min), and prolonged/fluctuating (>30 min or ≥3 episodes). The primary composite outcome included acute kidney injury, postoperative delirium, unplanned ICU admission within 48 h, and 30-day all-cause mortality. Associations were examined using multivariable logistic regression, and predictive performance was evaluated using ROC curves, calibration, bootstrap internal validation, and decision curve analysis.

**Results:**

A clear exposure-response relationship was observed across trajectory groups: the primary composite complication occurred in 13.4% of patients in the transient mild group, 20.8% in the moderate sustained group, and 30.7% in the prolonged/fluctuating group (p for trend <0.001). Compared with transient mild hypotension, adjusted odds ratios were 1.58 (95% CI 1.03–2.43) for moderate sustained and 2.42 (95% CI 1.54–3.80) for prolonged/fluctuating trajectories. Incorporating trajectory classification into a clinical model markedly improved discrimination (AUC 0.860 vs. 0.578), calibration, and net clinical benefit compared with conventional IOH measures alone.

**Conclusion:**

Distinct intraoperative hypotension trajectories derived from high-resolution arterial pressure data were strongly and independently associated with major postoperative complications and substantially enhanced predictive accuracy beyond standard IOH metrics. Trajectory-based hemodynamic profiling may support individualized blood pressure management and early perioperative risk stratification.

## Introduction

Intraoperative hypotension (IOH)—typically defined by an absolute mean arterial pressure (MAP) threshold of approximately 65 mmHg—is a frequent event during non-cardiac surgery; for example, a large community-based analysis found that 29% of cases accumulated at least 15 min with MAP < 65 mmHg (1, 2). IOH—most often delineated by MAP thresholds in the 60–70 mmHg range—has been associated with a broad spectrum of adverse outcomes in non-cardiac surgery, including acute kidney injury (AKI), myocardial injury, postoperative delirium, stroke, and death. Contemporary consensus statements and cohort studies further demonstrate that these risks are graded and strongly dependent on the cumulative duration of hypotension (3–5). A recent systematic review and meta-analysis reported that IOH was linked to significantly higher odds of 30-day mortality (OR 1.85), AKI (OR 2.69), myocardial infarction (OR 2.11), and postoperative delirium (OR 2.27) compared with non-hypotensive cases (6).

Both experimental and clinical evidence indicate that not only the severity but also the cumulative duration of IOH are critical determinants of organ hypoperfusion and ischemia. International consensus statements emphasize minimizing both the depth and length of hypotensive episodes (7), while large-scale data show that each additional minute of hypotension independently increases the risk of postoperative AKI (8). Furthermore, observational studies suggest that the prognostic impact of hypotension may differ depending on its timing—such as early post-induction versus later intraoperative phases—highlighting the relevance of both exposure duration and temporal context (9).

Although numerous studies have quantified intraoperative hypotension using nadir MAP, cumulative minutes below prespecified thresholds, area-under-the-threshold indices, or time-weighted average MAP, these approaches typically summarize exposure into a single aggregate metric. Such methods capture severity and duration, but they do not characterize the temporal organization of hypotensive events—such as clustering, recurrence, or evolving patterns—which may carry additional prognostic information (1). Group-based trajectory modeling (GBTM) therefore offers a complementary framework by identifying latent longitudinal patterns in high-resolution MAP data, integrating depth, duration, recurrence, and timing into distinct hemodynamic phenotypes rather than relying on a single summary value. Such an approach fails to distinguish between brief, self-limited episodes and prolonged or recurrent hypotension, which may have distinct pathophysiologic mechanisms and prognostic implications. Recent methodological advances—particularly group-based trajectory modeling (GBTM) and its multitrajectory extensions—allow the identification of latent hemodynamic phenotypes from longitudinal blood pressure data, capturing differences in duration, recurrence, and depth of IOH exposure (10, 11). While these techniques have shown promise in other perioperative contexts, their application to intraoperative blood pressure dynamics remains limited, and their prognostic value in high-risk surgical populations is largely unknown.

High-risk procedures such as major abdominal, urologic, and gynecologic surgeries are characterized by long operative times, substantial fluid shifts, and frequent hemodynamic instability, making them an optimal setting for studying IOH patterns. Accurate perioperative hemodynamic risk stratification in these patients could enable earlier intervention, individualized blood pressure targets, and improved postoperative outcomes.

In parallel, there has been growing interest in leveraging machine learning to predict perioperative hemodynamic instability and adverse outcomes from high-resolution monitoring data. Recent work has shown that hypotension prediction indices and other machine-learning methods can anticipate intraoperative hypotension and related complications (12, 13), while explainable machine-learning models have been developed for perioperative risk prediction in domains such as postoperative nausea and vomiting or acute kidney injury (14, 15). These studies underscore the potential of data-driven approaches to exploit continuous intraoperative signals for real-time risk stratification, but they have not specifically focused on characterizing longitudinal IOH trajectories as distinct hemodynamic phenotypes.

To address these gaps, we conducted a large retrospective cohort study of 789 consecutive adult patients undergoing major elective surgery with continuous invasive arterial pressure monitoring. Using GBTM applied to minute-by-minute MAP recordings, we identified distinct IOH trajectories and examined their associations with major postoperative complications. We further evaluated whether trajectory classification improved risk prediction beyond conventional clinical models, aiming to establish a framework for trajectory-informed intraoperative management strategies.

## Methods

### Study design and setting

This study was a retrospective, single-center cohort analysis conducted at The Affiliated Traditional Chinese Medicine Hospital, Southwest Medical University, a tertiary referral hospital in Southwest China. The investigation covered the period from January 2018 to December 2023 and included adult patients who underwent elective major abdominal, urologic, or gynecologic surgery under general anesthesia with continuous invasive arterial pressure monitoring. Because of the retrospective nature of the analysis, no patient contact or intervention was involved. All perioperative data were extracted from pre-existing electronic systems, and therefore this study posed no additional risk to participants. The research protocol was approved by the institutional ethics committee (Approval No. BY2025088), which waived the requirement for informed consent due to the retrospective design and anonymization of data.

### Participants

All adult patients (≥18 years) who met the inclusion criteria during the study period were consecutively enrolled to minimize selection bias. Inclusion criteria were: (1) elective major abdominal, urologic, or gynecologic surgery with invasive arterial blood pressure monitoring from induction to emergence; and (2) complete perioperative clinical information available in the anesthesia information system. Exclusion criteria included: (1) missing >5% of intraoperative arterial pressure data or >5 consecutive minutes of signal loss; (2) end-stage renal disease, advanced hepatic failure (Child–Pugh class C), or New York Heart Association class IV heart failure; (3) intraoperative death or aborted procedures; and (4) emergency surgery. All eligible cases were identified through a complete query of the anesthesia information management system (AIMS), ensuring comprehensive inclusion and eliminating sampling bias. No matching or prospective follow-up was performed because all data were derived from completed medical records.

### Variables and definitions

The primary exposure was intraoperative hypotension, defined as mean arterial pressure (MAP) < 65 mmHg, measured continuously via invasive arterial line. Outcome variables included a composite of (1) acute kidney injury (AKI, defined per KDIGO criteria), (2) postoperative delirium (confirmed using CAM-ICU and DSM-5 criteria), (3) unplanned ICU admission within 48 h, or (4) all-cause 30-day mortality. Secondary outcomes comprised reoperation within 7 days, surgical site or organ-space infection (CDC/NHSN criteria), ICU readmission within 7 days, and postoperative hospital length of stay. For the purposes of this study, outcomes were ascertained over prespecified postoperative windows: AKI and postoperative delirium were evaluated from the end of surgery through postoperative day 7 or hospital discharge (whichever occurred first); unplanned ICU admission within 48 h, ICU readmission within 7 days, and reoperation within 7 days were identified from time-stamped ICU and operating room records; 30-day all-cause mortality was determined from EMR problem lists, discharge summaries, and the institutional death registry; and postoperative hospital length of stay was defined as the number of days from the index surgery to hospital discharge.

### Data sources and measurement

All perioperative information was obtained from the hospital’s electronic medical records (EMR) and AIMS, which automatically capture minute-by-minute invasive MAP values. Data acquisition hardware and software remained uniform throughout the study period. Automated artifact detection algorithms identified erroneous waveforms, which were manually reviewed by two investigators blinded to outcomes. Short gaps (<5 min) were linearly interpolated, while longer missing segments led to case exclusion. Laboratory and outcome variables were physician-verified at data entry. All postoperative outcomes—including AKI, delirium, ICU admissions and readmissions, reoperations, infections, mortality, and length of stay—were ascertained exclusively from the hospital EMR using a combination of structured diagnosis fields, laboratory values, ICU flow sheets, and narrative discharge summaries; these outcome variables are not recorded in the AIMS, which is limited to intraoperative monitoring and anesthesia-related data. Diagnostic definitions (KDIGO, DSM-5, CDC/NHSN) ensured consistency across patients. The EMR and AIMS datasets were merged using unique patient identifiers and surgery timestamps, and linkage accuracy was verified by cross-checking overlapping variables such as procedure type, date, and anesthesia start time; concordance for these linkage fields exceeded 99%, indicating reliable matching between the two systems rather than duplication of outcome information. All outcome data were extracted for research purposes only after at least 30 postoperative days had elapsed for the last included surgery, ensuring a complete 30-day follow-up window for every patient in the cohort.

### Bias control

To address potential bias inherent in retrospective analyses, multiple strategies were implemented. First, consecutive inclusion of all eligible surgeries during the study period minimized selection bias. Second, standardized data collection from automated monitoring systems reduced measurement bias. Third, independent double verification of critical variables (blood loss, operative duration, outcome classification) and adjudication by blinded reviewers minimized misclassification. Finally, statistical adjustment for key clinical confounders (age, ASA class, comorbidities, intraoperative factors) reduced residual confounding.

### Study size

Because the study was retrospective and utilized existing electronic anesthesia records, all eligible cases during the five-year period were included. Nevertheless, the sample size was verified by *a priori* estimation using preliminary data, assuming a 20% baseline event rate and an odds ratio of 1.8 for major complications, which indicated that at least 720 cases were required to achieve 80% statistical power at *α* = 0.05. The final sample of 789 patients exceeded this threshold, ensuring robust analytic validity.

### Handling of quantitative variables

Continuous variables were summarized as mean ± standard deviation (SD) or median with interquartile range (IQR), depending on normality (assessed by Shapiro–Wilk test). Categorical variables were expressed as counts and percentages. For trajectory classification, hypotension duration and recurrence were grouped into clinically meaningful categories (<10 min, 10–30 min, and >30 min or ≥3 episodes) based on prior literature and the observed distribution within the dataset.

### Statistical analysis

Statistical analyses were performed using R software (version 4.2.2; R Foundation for Statistical Computing, Vienna, Austria). Group-based trajectory modeling (GBTM) was applied to minute-by-minute mean arterial pressure (MAP) data using the traj package to identify latent intraoperative hypotension patterns. MAP was modeled as a function of absolute intraoperative time (minutes since induction) using third-order (cubic) polynomial terms of time within each latent class, assuming a censored normal distribution for the continuous MAP outcome. We initially fitted models with two to five trajectory classes and compared them using the Bayesian Information Criterion (BIC), mean posterior class-membership probabilities, minimum group size, and clinical interpretability of the resulting patterns. The final three-class solution was selected because it had the lowest BIC among clinically plausible models, mean posterior probabilities ≥0.80 for all classes, and no trajectory group comprising <10% of the cohort; procedures lasting longer than 180 min were truncated at 180 min, whereas shorter procedures contributed all available MAP observations without padding, as the GBTM framework accommodates trajectories of unequal length. For comparisons across the three trajectory groups, overall *p*-values in Table 1 were derived from global Chi-square tests for categorical outcomes (or Kruskal–Wallis tests for non-normally distributed continuous variables), whereas the p for trend values were calculated by treating the trajectory category as an ordinal variable (Trajectory A = 1, B = 2, C = 3) in logistic regression and testing for a monotonic increase in complication rates.

**Table 1:** Incidence of postoperative complications across intraoperative hypotension trajectory patterns (*n* = 789).

Postoperative Outcome	Total (*n* = 789)	Trajectory A (*n* = 253)	Trajectory B (*n* = 308)	Trajectory C (*n* = 228)	*p*-value	*p* for trend
Primary composite complication*, *n* (%)	168 (21.3)	34 (13.4)	64 (20.8)	70 (30.7)	<0.001	<0.001
Acute kidney injury (KDIGO), *n* (%)	90 (11.4)	15 (5.9)	34 (11.0)	41 (18.0)	<0.001	<0.001
Postoperative delirium, *n* (%)	54 (6.8)	9 (3.6)	19 (6.2)	26 (11.4)	0.001	<0.001
ICU admission within 48 h, *n* (%)	126 (16.0)	25 (9.9)	49 (15.9)	52 (22.8)	<0.001	<0.001
30-day all-cause mortality, *n* (%)	18 (2.3)	2 (0.8)	5 (1.6)	11 (4.8)	0.008	0.004
Secondary complications, *n* (%)						
Reoperation within 7 days	22 (2.8)	4 (1.6)	7 (2.3)	11 (4.8)	0.042	0.019
Surgical site or organ space infection	104 (13.2)	23 (9.1)	38 (12.3)	43 (18.9)	0.007	0.003
Length of postoperative hospital stay, days						
Median (IQR)	10 (8–14)	9 (7–12)	10 (8–13)	12 (9–17)	<0.001	<0.001
ICU readmission within 7 days	16 (2.0)	2 (0.8)	4 (1.3)	10 (4.4)	0.010	0.006

ICU, intensive care unit; IQR, interquartile range; KDIGO, kidney disease: improving global outcomes; MAP, mean arterial pressure.

Trajectory A, transient mild hypotension (MAP <65 mmHg for <10 min); Trajectory B, moderate sustained hypotension (MAP <65 mmHg for 10–30 min); Trajectory C, prolonged or fluctuating hypotension (MAP <65 mmHg for >30 min or ≥3 episodes). Primary composite complication includes any of the following within 30 days: AKI, postoperative delirium, ICU admission, or death. Complication definitions were based on KDIGO (AKI), CAM-ICU/DSM-5 (delirium), and institutional ICU admission protocols. p-values were derived from Chi-square or Fisher’s exact tests for categorical variables and Kruskal–Wallis test for non-normally distributed continuous variables; p for trend was obtained by treating the trajectory category as an ordinal variable (A = 1, B = 2, C = 3) and testing for a monotonic increase in outcome rates across trajectories.

Independent predictors of the primary composite outcome were determined by multivariable logistic regression, including variables that were clinically relevant or had a univariable *p* < 0.10; multicollinearity was assessed using variance inflation factors, with VIF > 5 considered exclusionary. Model discrimination was assessed by the area under the receiver operating characteristic curve (AUC) and calibration by the Hosmer–Lemeshow goodness-of-fit test and calibration plots. To assess the incremental predictive value of hypotension trajectory grouping, three nested models were compared: Model 1 (clinical variables only), Model 2 (clinical variables plus trajectory group), and Model 3 (trajectory group only). In additional exploratory analyses, we also constructed logistic regression models in which conventional IOH exposure metrics—nadir MAP, cumulative minutes with MAP <65 mmHg, and the time-weighted average (TWA) MAP <65 mmHg—were entered as predictors, either alone or in place of the trajectory variable; the performance of these conventional-metric models is summarized in Supplementary Table S1 to facilitate direct comparison with the trajectory-based models. Internal validation was performed using bootstrap resampling (1,000 replicates) to adjust for optimism. Missing data (<5% for all variables) were imputed using multiple imputation with chained equations under the missing-at-random assumption. Decision curve analysis (DCA) and clinical impact curves (CIC) were used to quantify clinical utility and net benefit across risk thresholds. All tests were two-tailed, and *p* < 0.05 was considered statistically significant.

## Results

### Baseline and intraoperative characteristics by hypotension trajectory patterns

**Figure 1:**
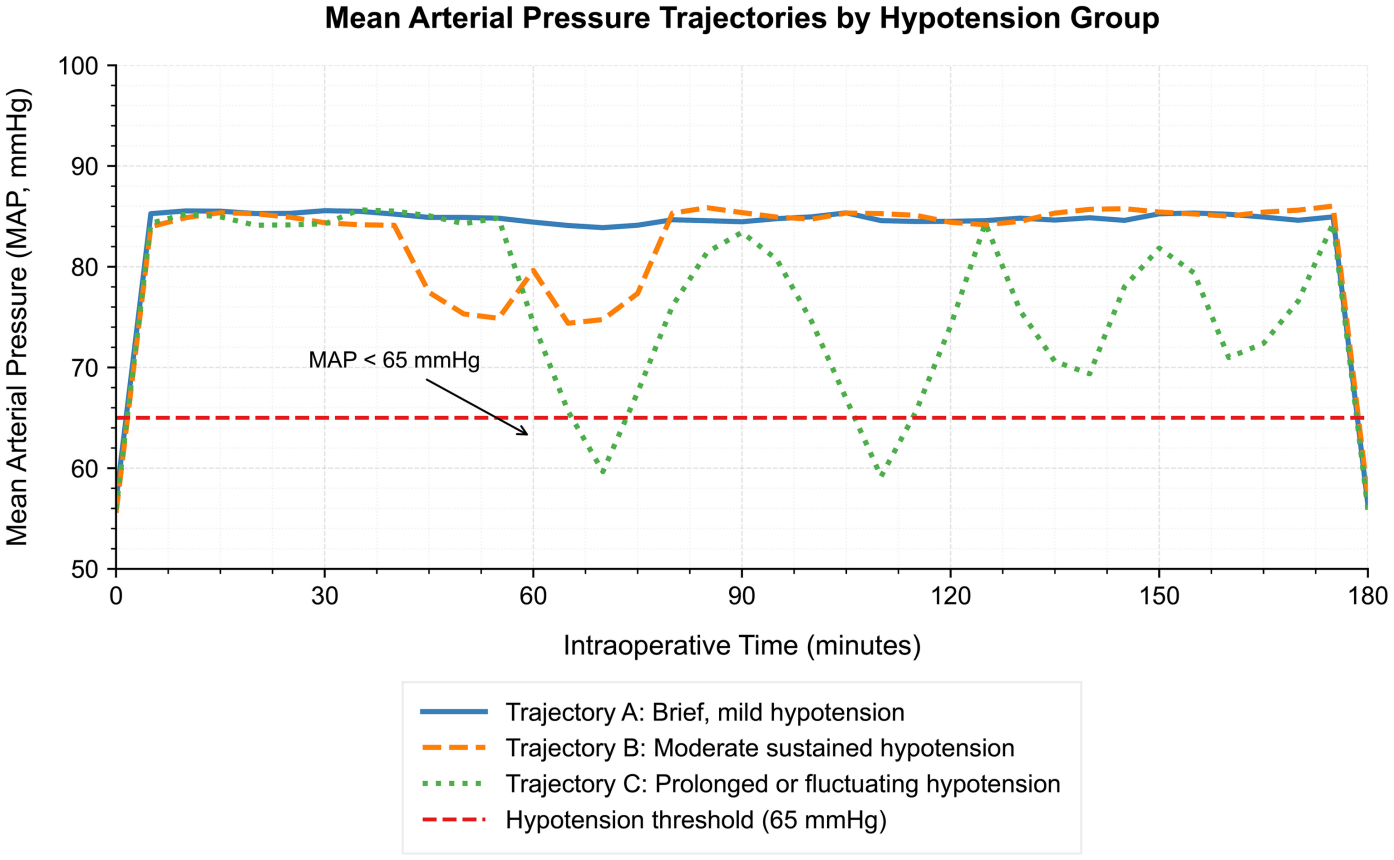
Mean arterial pressure (MAP) trajectories during surgery, stratified by intraoperative hypotension patterns. Patients were classified into three predefined trajectory groups based on the cumulative duration and pattern of MAP <65 mmHg: Trajectory A, transient mild hypotension (<10 min), Trajectory B, moderate sustained hypotension (10–30 min), and Trajectory C, prolonged or fluctuating hypotension (>30 min or multiple episodes). Each curve depicts the group-averaged MAP at each intraoperative time point, illustrating distinct hypotensive exposure profiles. The dashed red horizontal line indicates the clinical hypotension threshold (MA*P* = 65 mmHg).

Baseline demographic and intraoperative characteristics of the study cohort, stratified by intraoperative hypotension trajectory patterns, are summarized in Table 2. Among the 789 patients, 253 (32.1%) were classified as Trajectory A (brief, mild hypotension), 308 (39.0%) as Trajectory B (moderate sustained hypotension), and 228 (28.9%) as Trajectory C (prolonged or fluctuating hypotension). As detailed in Table 2, baseline and intraoperative characteristics differed significantly across the three trajectory groups. In general, patients in Trajectory C were older, had more comorbidities, and experienced longer and more hemodynamically demanding operations, whereas Trajectory A included younger patients with fewer comorbidities. The temporal patterns of mean arterial pressure (MAP) for each trajectory group are depicted in Figure 1. Trajectory A exhibited stable MAP values with only transient, mild decreases below the hypotension threshold (65 mmHg). Trajectory B demonstrated sustained moderate hypotension lasting 10–30 min, while Trajectory C showed repeated or prolonged episodes of hypotension, often markedly below the threshold, reflecting greater intraoperative hemodynamic instability.

**Table 2:** Baseline and intraoperative characteristics of patients stratified by intraoperative hypotension trajectory patterns (*n* = 789).

Characteristics	Total (*n* = 789)	Trajectory A (*n* = 253)	Trajectory B (*n* = 308)	Trajectory C (*n* = 228)	*p*-value
Age, years, mean ± SD	65.2 ± 11.8	62.1 ± 11.3ᵃ	66.7 ± 10.9ᵇ	68.3 ± 12.0ᵇ	<0.001
Male sex, *n* (%)	462 (58.6)	141 (55.7)ᵃ	178 (57.8)ᵃ	143 (62.7)ᵃ	0.287
BMI, kg/m^2^, mean ± SD	23.6 ± 3.9	24.1 ± 3.6ᵃ	23.3 ± 3.8ᵇ	23.2 ± 4.1ᵇ	0.014
ASA classification III–IV, *n* (%)	402 (51.0)	104 (41.1)ᵃ	165 (53.6)ᵇ	133 (58.3)ᵇ	<0.001
Hypertension, *n* (%)	378 (47.9)	94 (37.2)ᵃ	150 (48.7)ᵇ	134 (58.8)ᶜ	<0.001
Diabetes mellitus, *n* (%)	205 (26.0)	53 (20.9)ᵃ	79 (25.6)ᵃᵇ	73 (32.0)ᵇ	0.018
Coronary artery disease, *n* (%)	122 (15.5)	27 (10.7)ᵃ	45 (14.6)ᵃᵇ	50 (21.9)ᵇ	0.004
Preoperative hemoglobin, g/L	127.2 ± 16.5	129.8 ± 14.2ᵃ	126.1 ± 15.7ᵃᵇ	124.4 ± 17.9ᵇ	0.009
Surgery type, *n* (%)	—	—	—	—	0.041
Hepatobiliary/pancreatic	212 (26.9)	58 (22.9)ᵃ	86 (27.9)ᵃᵇ	68 (29.8)ᵇ	—
Gastrointestinal	226 (28.6)	82 (32.4)ᵃ	85 (27.6)ᵃᵇ	59 (25.9)ᵇ	—
Urologic	188 (23.8)	68 (26.9)ᵃ	70 (22.7)ᵃ	50 (21.9)ᵃ	—
Gynecologic	99 (12.6)	34 (13.4)ᵃ	40 (13.0)ᵃ	25 (11.0)ᵃ	—
Anesthesia type	—	—	—	—	0.153
General anesthesia	713 (90.4)	231 (91.3)ᵃ	275 (89.3)ᵃ	207 (90.8)ᵃ	—
Intraoperative duration, min (IQR)	175 (130–225)	150 (115–200)ᵃ	180 (140–230)ᵇ	195 (150–250)ᶜ	<0.001
Estimated blood loss, mL (IQR)	320 (180–650)	260 (150–500)ᵃ	350 (200–680)ᵇ	420 (250–750)ᶜ	<0.001
Ephedrine/norepinephrine use, *n* (%)	498 (63.1)	132 (52.2)ᵃ	207 (67.2)ᵇ	159 (69.7)ᵇ	<0.001
Total fluid input, mL (IQR)	2,350 (1900–3,000)	2,100 (1700–2,600)ᵃ	2,400 (1950–3,100)ᵇ	2,600 (2100–3,300)ᶜ	<0.001

Values with different superscript letters (a, b, c) within the same row differ significantly at *p* < 0.05 based on Bonferroni-adjusted pairwise comparisons. BMI, body mass index; ASA, American Society of Anesthesiologists; IQR, interquartile range; SD, standard deviation. Trajectory A, transient mild IOH; Trajectory B, moderate sustained IOH; Trajectory C, prolonged/fluctuating IOH. Values that do not share a superscript letter differ significantly at *p* < 0.05 based on Bonferroni-adjusted post-hoc pairwise comparisons.

### Postoperative complications by hypotension trajectory patterns

The incidence of postoperative complications differed significantly across intraoperative hypotension trajectory groups (Table 1). The primary composite complication occurred in 21.3% of patients overall, with a stepwise increase from 13.4% in Trajectory A to 20.8% in Trajectory B and 30.7% in Trajectory C (*p* < 0.001 for both overall comparison and trend). Among individual components, the rates of acute kidney injury (AKI), postoperative delirium, ICU admission within 48 h, and 30-day all-cause mortality all increased progressively from Trajectory A to C (all p for trend ≤ 0.004). Notably, AKI incidence was 18.0% in Trajectory C, more than threefold higher than in Trajectory A (5.9%). Secondary complications showed similar patterns. The frequency of reoperation within 7 days, surgical site or organ space infection, and ICU readmission within 7 days was highest in Trajectory C. Median postoperative hospital stay was significantly longer in Trajectory C (12 days, IQR 9–17) compared with Trajectories A and B (9 and 10 days, respectively; *p* < 0.001 for trend). Overall, a clear dose–response relationship was observed, with more severe or prolonged hypotension trajectories associated with higher complication rates and longer postoperative recovery.

### Independent predictors of primary composite complications

Multivariable logistic regression analysis identified intraoperative hypotension trajectory as an independent predictor of the primary composite complication (Table 3). Compared with Trajectory A, patients in Trajectory B had a 58% higher adjusted odds of developing the primary composite complication (adjusted OR 1.58, 95% CI 1.03–2.43, *p* = 0.035), while those in Trajectory C had more than double the risk (adjusted OR 2.42, 95% CI 1.54–3.80, *p* < 0.001), with a significant dose–response relationship (p for trend < 0.001). Other independent predictors included older age (per 10-year increase: adjusted OR 1.19, 95% CI 1.04–1.37, *p* = 0.012), ASA classification III–IV (adjusted OR 1.61, 95% CI 1.14–2.29, *p* = 0.007), coronary artery disease (adjusted OR 1.77, 95% CI 1.10–2.85, *p* = 0.019), preoperative hemoglobin <120 g/L (adjusted OR 1.49, 95% CI 1.06–2.09, *p* = 0.022), intraoperative duration >180 min (adjusted OR 1.42, 95% CI 1.02–1.98, *p* = 0.039), and estimated blood loss >500 mL (adjusted OR 1.72, 95% CI 1.17–2.53, *p* = 0.006). Vasopressor use showed a non-significant association (*p* = 0.112). These findings indicate that more severe or prolonged intraoperative hypotension trajectories remain strong, independent risk factors for major postoperative complications, even after adjusting for patient comorbidities and intraoperative variables.

**Table 3:** Multivariable logistic regression analysis for primary composite complications (*n* = 789).

Variable	Adjusted OR	95% CI	*p*-value	*p* for trend*
Intraoperative hypotension trajectory				<0.001
Trajectory A	Reference	—	—	
Trajectory B	1.58	1.03–2.43	0.035	
Trajectory C	2.42	1.54–3.80	<0.001	
Age, per 10-year increase	1.19	1.04–1.37	0.012	—
ASA classification III–IV	1.61	1.14–2.29	0.007	—
Coronary artery disease	1.77	1.10–2.85	0.019	—
Preoperative hemoglobin <120 g/L	1.49	1.06–2.09	0.022	—
Intraoperative duration >180 min	1.42	1.02–1.98	0.039	—
Estimated blood loss >500 mL	1.72	1.17–2.53	0.006	—
Vasopressor use (ephedrine/norepinephrine)	1.29	0.94–1.78	0.112	—

OR, odds ratio; CI, confidence interval; ASA, American Society of Anesthesiologists; Hb, hemoglobin; MAP, mean arterial pressure. Trajectory A, transient mild hypotension (MAP <65 mmHg for <10 min); Trajectory B, moderate sustained hypotension (MAP <65 mmHg for 10–30 min); Trajectory C, prolonged or fluctuating hypotension (MAP <65 mmHg for >30 min or ≥3 episodes). #Primary composite complication includes any of the following within 30 days: acute kidney injury, postoperative delirium, ICU admission, or death. Variables included in the multivariable model were selected based on clinical relevance and a univariate *p*-value <0.10. The model was adjusted for all variables listed. *p for trend was calculated by treating the trajectory category as an ordinal variable.

### Intraoperative blood pressure profiles by hypotension trajectory patterns

Intraoperative blood pressure characteristics differed markedly among the three hypotension trajectory groups (Table 4). Patients in Trajectory C had the lowest mean baseline MAP (89.4 ± 11.1 mmHg) and the lowest mean intraoperative nadir MAP (56.4 ± 7.1 mmHg), compared with higher values in Trajectories A and B (both *p* < 0.001). The number of hypotensive episodes increased stepwise from Trajectory A to C (median 1 vs. 3 vs. 6 episodes; *p* < 0.001), paralleled by longer cumulative hypotension duration (5 vs. 20 vs. 45 min; *p* < 0.001) and longer single-episode durations. The time-weighted average MAP below 65 mmHg was markedly elevated in Trajectory C (5.3 ± 2.1 mmHg) compared with Trajectory B (2.6 ± 1.3 mmHg) and Trajectory A (0.9 ± 0.6 mmHg; *p* < 0.001), indicating more prolonged and severe hypotensive exposure. Vasopressor bolus requirements followed a similar gradient, with the highest median doses in Trajectory C [3 (IQR 2–5)] and the lowest in Trajectory A [1 (IQR 0–2); *p* < 0.001]. These findings confirm that the predefined trajectory groups capture distinct patterns of intraoperative hemodynamic instability, with Trajectory C representing the most severe and sustained hypotension profile.

**Table 4:** Intraoperative blood pressure characteristics across hypotension trajectory patterns (*n* = 789).

Parameter	Total (*n* = 789)	Trajectory A (*n* = 253)	Trajectory B (*n* = 308)	Trajectory C (*n* = 228)	*p*-value
Baseline MAP, mmHg, mean ± SD	91.5 ± 10.4	93.1 ± 10.1	91.0 ± 9.9	89.4 ± 11.1	0.006
Lowest intraoperative MAP, mmHg, mean ± SD	61.2 ± 7.8	65.8 ± 6.2	60.7 ± 6.8	56.4 ± 7.1	<0.001
Number of hypotensive episodes (MAP <65 mmHg)	3 (1–5)	1 (1–2)	3 (2–5)	6 (4–9)	<0.001
Cumulative hypotension time, min, median (IQR)	18 (8–36)	5 (3–8)	20 (12–34)	45 (30–68)	<0.001
Longest single hypotensive episode, min	9 (4–18)	3 (2–5)	10 (6–18)	20 (12–32)	<0.001
Time-weighted average MAP <65 mmHg, mmHg	2.8 ± 1.9	0.9 ± 0.6	2.6 ± 1.3	5.3 ± 2.1	<0.001
Vasopressor bolus doses, n, median (IQR)	2 (1–4)	1 (0–2)	2 (1–4)	3 (2–5)	<0.001

MAP, mean arterial pressure; SD, standard deviation; IQR, interquartile range. Trajectory A, transient mild hypotension (MAP <65 mmHg for <10 min); Trajectory B, moderate sustained hypotension (MAP <65 mmHg for 10–30 min); Trajectory C = prolonged or fluctuating hypotension (MAP <65 mmHg for >30 min or ≥3 episodes). Baseline MAP was defined as the average of the three measurements prior to anesthesia induction. Hypotensive episodes were defined as any 5-min interval with MAP <65 mmHg. Time-weighted average MAP was calculated as the area under the threshold (65 mmHg) divided by total anesthesia time. *p*-values were calculated using ANOVA or Kruskal–Wallis tests for continuous variables and Chi-square tests for categorical variables.

### Predictive performance and clinical utility of the models

**Figure 2:**
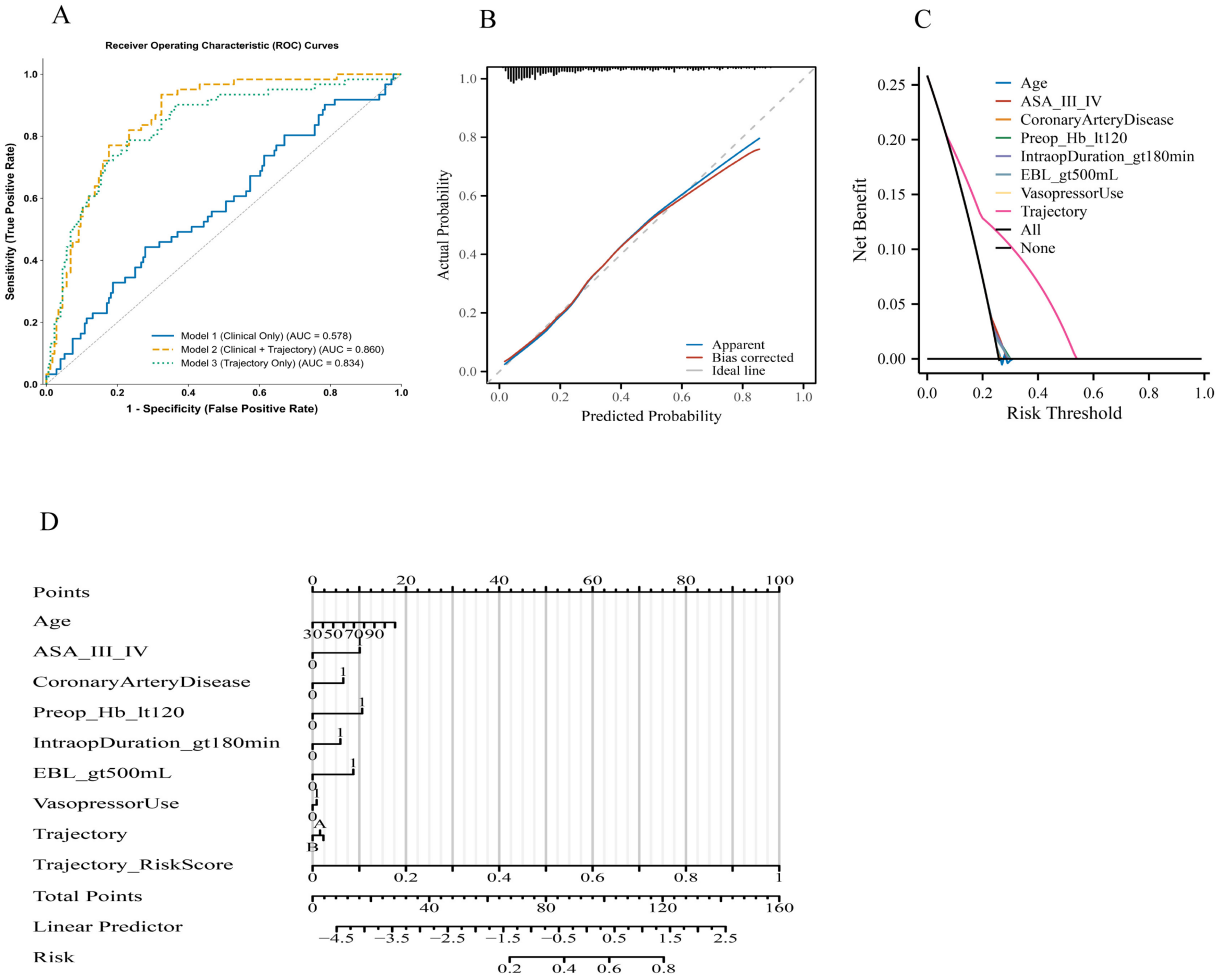
Discriminative, calibration, and clinical utility performance of predictive models for primary composite complications. **(A)** Receiver operating characteristic (ROC) curves comparing three models: Model 1 (clinical only), Model 2 (clinical + trajectory), and Model 3 (trajectory only). Area under the curve (AUC) values indicate highest discrimination for Model 2, followed by Model 3, and lowest for Model 1. **(B)** Calibration plot for Model 2 showing agreement between predicted and observed probabilities in the apparent (blue) and bias-corrected (red) curves against the ideal line (grey). **(C)** Decision curve analysis comparing three approaches: the clinical-only model, the trajectory-only model, and the combined model. Each curve represents the net benefit across threshold probabilities. The “All” and “None” reference lines correspond to treating all or none of the patients. The combined model consistently demonstrates higher net benefit between 0.10 and 0.35, while the trajectory-only and clinical-only models diverge modestly but remain above the treat-all strategy. Label descriptions have been clarified to improve readability. **(D)** Clinical impact curves (CIC) for Model 2, displaying the number of patients classified as high risk and the corresponding true positives at each risk threshold. Notes: Primary composite complication includes any of the following within 30 days: acute kidney injury, postoperative delirium, ICU admission, or death. Curves and metrics were generated using internal bootstrap validation with 1,000 resamples.

The comparative performance metrics of the three predictive models for primary composite complications are summarized in Table 5 and illustrated in Figure 2. Discrimination, assessed by the area under the ROC curve (AUC), was highest for Model 2 (Clinical + Trajectory) with an AUC of 0.860 (95% CI, 0.832–0.888), followed by Model 3 (Trajectory Only) at 0.834 (95% CI, 0.803–0.862), and lowest for Model 1 (Clinical Only) at 0.578 (95% CI, 0.531–0.624) (Figure 2A). Sensitivity and specificity were balanced in Models 2 and 3, with Model 2 achieving the highest sensitivity (77.0%) and a specificity of 76.2%, while Model 1 showed lower sensitivity (64.2%) and markedly reduced specificity (49.4%). Negative predictive values were highest for Model 2 (90.5%) and Model 3 (88.2%), indicating superior ability to rule out complications compared with Model 1 (79.8%). Calibration analysis of Model 2 (Figure 2B) demonstrated close alignment between predicted and observed probabilities, with both apparent and bias-corrected curves closely following the ideal line. Goodness-of-fit was supported by non-significant Hosmer–Lemeshow *p*-values for all models (*p* > 0.05), and Brier scores were lowest for Model 2 (0.173), reflecting superior overall accuracy. Decision curve analysis (Figure 2C) revealed that Model 2 provided the greatest net clinical benefit across a broad range of risk thresholds, consistently outperforming both Model 1 and Model 3 as well as the “treat all” and “treat none” strategies. To enhance clarity, the interpretation of each curve in Figure 2C has been elaborated in the legend. In particular, the magenta line corresponds to the combined model, the blue and green lines represent the trajectory-only and clinical-only models, respectively, and the black and gray dashed lines denote treat-all and treat-none strategies. This clarification allows the reader to more easily distinguish the net benefit patterns without altering the original figure. Clinical impact curves (Figure 2D) for Model 2 further illustrated its practical utility, showing a higher number of true positives among patients classified as high risk, particularly at clinically relevant thresholds. Collectively, these findings confirm that incorporating intraoperative hypotension trajectory patterns into a clinical model substantially improves discrimination, calibration, and potential clinical usefulness compared with a model based solely on clinical variables.

**Table 5:** Predictive performance of three models for primary composite complications# (*n* = 789).

Performance metric	Model 1: Clinical only	Model 2: Clinical + trajectory	Model 3: Trajectory only
**AUC (95% CI)**	0.578 (0.531–0.624)	**0.860 (0.832–0.888)**	0.834 (0.803–0.862)
Sensitivity (%)	64.2 (58.1–70.0)	**77.0 (71.5–82.0)**	70.6 (64.8–75.9)
Specificity (%)	49.4 (44.1–54.7)	76.2 (71.3–80.6)	**76.8 (72.0–81.0)**
PPV (%)	30.7 (26.1–35.7)	**53.0 (47.6–58.4)**	51.4 (45.9–56.9)
NPV (%)	79.8 (75.3–83.7)	**90.5 (87.1–93.1)**	88.2 (84.5–91.2)
Accuracy (%)	53.2 (48.8–57.6)	**76.4 (72.6–79.8)**	75.2 (71.4–78.8)
Hosmer–Lemeshow *p*	0.183	**0.412**	0.368
Brier score	0.245	**0.173**	0.183

ROC, receiver operating characteristic; AUC, area under the ROC curve; PPV, positive predictive value; NPV, negative predictive value. #Primary composite complication includes any of the following within 30 days: acute kidney injury, postoperative delirium, ICU admission, or death. Performance metrics were derived from internal validation using bootstrapping with 1,000 resamples, with the optimal cut-off determined by the Youden index. Hosmer–Lemeshow *p* > 0.05 indicates good calibration, and a lower Brier score reflects better overall model accuracy. Bold values indicate the best performance among the three models for each metric.

To complement these primary models, we also fitted exploratory logistic regression models incorporating conventional IOH exposure metrics (nadir MAP, cumulative minutes with MAP <65 mmHg, and TWA MAP <65 mmHg) in place of the trajectory classification. Their discrimination, calibration, and decision-analytic characteristics are reported in Supplementary Table S1, allowing readers to directly compare traditional IOH summary measures with trajectory-based classification.

## Discussion

In this retrospective cohort of 789 high-risk surgical patients, we identified three distinct intraoperative hypotension (IOH) trajectories—transient mild, moderate sustained, and prolonged/fluctuating—each demonstrating a graded association with major postoperative complications. Compared with the conventional binary definition of IOH, our trajectory-based approach provided markedly greater predictive accuracy, improving both discrimination (AUC 0.860 vs. 0.578) and calibration when combined with standard clinical variables.

Our findings extend the existing literature by shifting the focus from static mean arterial pressure (MAP) thresholds to dynamic, longitudinal characterization of intraoperative hemodynamics. Prior evidence supports a graded exposure–response relationship in which both the depth and cumulative duration of IOH independently contribute to organ injury: contemporary consensus guidelines recommend minimizing both the magnitude and duration of MAP depression (3); a 2022 narrative review reported that deeper and longer hypotensive episodes are associated with increased risks of postoperative myocardial injury and mortality (4); and a 2023 meta-analysis confirmed significantly higher odds of acute kidney injury (AKI), myocardial infarction, and mortality in hypotensive patients (6). Consistent with these observations, our prolonged/fluctuating trajectory—characterized by extended MAP depression or multiple recurrent episodes—was associated with more than twice the adjusted odds of major complications compared with transient mild hypotension. This magnitude of association is aligned with multi-center evidence showing that each additional minute with MAP <65 mmHg confers a dose-dependent increase in the risk of postoperative AKI and myocardial injury (16, 17). Importantly, our classification framework captures recurrence, a dimension largely neglected in prior research. Repeated hypotensive episodes often reflect unresolved intraoperative pathophysiology—such as ongoing blood loss, excessive anesthetic depth with vasodilation, or impaired autonomic regulation—rather than isolated measurement error (7). Experimental and preclinical studies further suggest that cyclic hypoperfusion–reoxygenation provokes more pronounced oxidative and inflammatory responses than sustained ischemia of equivalent duration, which may help explain the elevated complication rates observed in the prolonged/fluctuating group (18, 19).

The strong predictive performance of the trajectory-augmented model has practical implications. Current guidelines recommend maintaining intraoperative MAP at or above 60–65 mmHg but offer limited evidence-based direction regarding acceptable cumulative exposure or recurrence thresholds, leaving a gap in temporal dosing metrics for hypotension (20, 21). Our results suggest that incorporating trajectory classification into intraoperative decision-support platforms could enable real-time identification of high-risk hemodynamic patterns and trigger targeted interventions such as preload optimization, adjustment of anesthetic depth, or vasopressor administration. Operationally, such a system could apply a pre-trained trajectory model at the bedside by updating each patient’s class-membership probabilities in near real time as MAP data accrue—for example, recalculating posterior probabilities every 5–10 min over the first 60–90 min of surgery and flagging patients whose probability of belonging to a prolonged/fluctuating trajectory exceeds a prespecified threshold (e.g., ≥0.70), while still allowing reclassification if subsequent hemodynamics stabilize.

In high-risk noncardiac surgeries—such as major abdominal, urologic, and gynecologic procedures—recent clinical trials have demonstrated the feasibility and clinical utility of individualized blood pressure targets and structured fluid–vasopressor strategies: for instance, large multicenter studies in major gastrointestinal surgery evaluated cardiac-output–guided fluid therapy with low-dose inotropes, while a bicentric randomized pilot demonstrated the practicality of tailoring intraoperative MAP to a patient’s preoperative nighttime baseline (22, 23). In the present single-center study, the three IOH trajectories should therefore be interpreted primarily as data-driven hemodynamic phenotypes and a proof-of-principle classifier rather than fixed universal categories; in other institutions, the same modeling framework could be retrained on local MAP time series, potentially yielding different numbers or shapes of trajectories while preserving the core concept of trajectory-informed IOH risk stratification.

Another important observation is that trajectory classification outperformed established predictors such as ASA physical status and baseline comorbidities in terms of both discrimination and calibration. While preoperative risk scores remain valuable, they do not capture the dynamic nature of intraoperative physiology. Integrating static preoperative factors with dynamic intraoperative variables aligns with the broader shift toward personalized, data-driven perioperative care: recent informatics frameworks combine pre- and intraoperative data streams for complication prediction and communication, and systematic reviews in anesthesiology highlight the promise of such multimodal, continuously updated models (24, 25). Building on this work, several recent studies have applied machine-learning approaches directly to perioperative monitoring data, including systematic evaluation of hypotension prediction indices and other algorithms for forecasting IOH (12), deep-learning models for predicting intraoperative hypotension from non-invasive signals (13), and explainable machine-learning tools for predicting postoperative nausea and vomiting or surgery-related acute kidney injury (14, 15). Our trajectory-based approach is conceptually complementary to these efforts: rather than generating a point prediction of a future event, it uses high-resolution MAP time series to identify latent IOH phenotypes that encode duration, depth, timing, and recurrence. In our cohort, decision curve analysis demonstrated that such integration yields meaningful net clinical benefit across a range of treatment thresholds, reinforcing the potential value of trajectory-informed monitoring in both research and practice.

Our study also addresses an analytical gap. Most large-scale IOH studies encode MAP exposure as a binary or cumulative-time variable, approaches that may obscure latent heterogeneity in exposure–response relationships; recent data-driven analyses show that the definition chosen (e.g., cumulative minutes below a MAP threshold vs. nadir depth) can substantially influence observed associations with outcomes (26). This observation is consistent with prior work by Vernooij et al. (27), who systematically compared multiple IOH modeling strategies—including presence, depth, duration, and area-under-the-threshold metrics—and demonstrated that both the blood pressure threshold and the analytic expression of IOH (e.g., nadir, cumulative duration, or TWA) can materially alter estimated associations with postoperative myocardial injury and acute kidney injury. In our cohort, additional models based on nadir MAP, cumulative hypotension time, or TWA MAP <65 mmHg (Supplementary Table S1) allow direct comparison of these conventional summary metrics with trajectory-based classification. Whereas conventional measures capture specific dimensions of IOH exposure, the trajectory-based approach used in the present study focuses on the temporal organization of hypotensive episodes—integrating timing, duration, depth, and recurrence into latent patterns—which may offer a complementary framework for characterizing IOH in both research and clinical decision-support settings. Group-based trajectory modeling (GBTM), though widely applied in other biomedical contexts, has rarely been used to characterize intraoperative blood pressure patterns.

Several limitations should be noted. First, the single-center design may limit generalizability; institutional practice patterns, patient demographics, and monitoring technology could affect trajectory distributions. Second, despite stringent artifact detection and waveform review, invasive MAP readings remain susceptible to calibration error and transducer malposition, although such errors are unlikely to produce systematic bias between groups. Third, residual confounding is possible given the observational nature of the study, despite adjustment for multiple perioperative variables. In addition, although major complications such as AKI, delirium, ICU admission, and mortality are routinely captured in the EMR for clinical and administrative reasons, we cannot entirely exclude under-ascertainment of less severe or transient events that may have been documented only in anesthesia narratives. Fourth, our IOH definition (MAP <65 mmHg) follows consensus recommendations but may not be optimal for all patients, particularly those with chronic hypertension who might require higher perfusion pressures. Future work should explore whether trajectory thresholds tailored to baseline physiology improve predictive utility. Finally, while our thresholds for trajectory classification were prespecified based on prior evidence, alternative schemes incorporating nadir depth, recovery slope, or intraoperative timing could yield further insights. Notably, recent evidence indicates that post-induction hypotension may have different prognostic implications than hypotension occurring later in surgery: in a 14,210-patient cohort, hypotension confined to the induction–incision interval was not associated with 30- or 90-day mortality, whereas IOH occurring later was, highlighting the importance of timing (28). Future studies should examine whether real-time modification of IOH trajectories through protocolized hemodynamic optimization translates into improved outcomes.

In conclusion, intraoperative hypotension trajectories derived from minute-by-minute MAP recordings provide independent and incremental prognostic information for major postoperative complications in high-risk surgical patients. Our findings support a shift from binary IOH definitions toward dynamic, trajectory-based risk assessment. Embedding such approaches into perioperative monitoring systems may enable earlier intervention, more individualized blood pressure targets, and ultimately improved surgical outcomes.

## Data Availability

The original contributions presented in the study are included in the article/supplementary material, further inquiries can be directed to the corresponding author.
