# Supplementary material for: Intraoperative hypotension trajectories and their predictive value for major postoperative complications: a retrospective cohort study
Source: Front Med (Lausanne). 2026 Jan 14;12:1739832. doi: 10.3389/fmed.2025.1739832 (PMC12847311; doi:10.3389/fmed.2025.1739832)
Supplement: Supplementary file 1 [file Table_1.DOCX]

**Supplementary Table S1.** Discrimination, Calibration, and Clinical Utility of Logistic Regression Models Using Conventional IOH Metrics (n = 789)

| **Performance Metric** | **Model A: Nadir MAP Only** | **Model B: Cumulative Minutes MAP <65 mmHg** | **Model C: TWA MAP <65 mmHg** |
| --- | --- | --- | --- |
| **AUC (95% CI)** | 0.612 (0.566–0.657) | 0.645 (0.600–0.689) | 0.662 (0.618–0.705) |
| **Sensitivity (%)** | 58.3 (52.1–64.3) | 61.9 (55.7–67.8) | 64.1 (58.0–69.8) |
| **Specificity (%)** | 56.7 (51.4–61.8) | 60.3 (55.0–65.3) | 61.6 (56.3–66.6) |
| **PPV (%)** | 29.1 | 32.7 | 33.9 |
| **NPV (%)** | 81.0 | 83.5 | 84.1 |
| **Accuracy (%)** | 57.1 | 60.8 | 62.2 |
| **Hosmer–Lemeshow p** | 0.231 | 0.287 | 0.301 |
| **Brier Score** | 0.237 | 0.224 | 0.218 |
| **Net Clinical Benefit (DCA)** | Low benefit across all thresholds; inferior to trajectory models | Moderate benefit at mid-range thresholds | Moderate benefit but still inferior to Model 2 (trajectory + clinical) |

**Note:** This table summarizes the predictive performance of logistic regression models incorporating conventional intraoperative hypotension (IOH) metrics: Model A: nadir intraoperative MAP; Model B: cumulative minutes with MAP <65 mmHg; Model C: time-weighted average (TWA) MAP <65 mmHg. All performance metrics were derived using 1,000 bootstrap resamples. These models were constructed to allow direct comparison with the trajectory-based models presented in Table 5. Across all three conventional IOH metrics, discrimination (AUC 0.612–0.662), calibration (Brier scores 0.218–0.237), and decision-analytic performance were consistently inferior to the trajectory-enhanced model (Model 2 in Table 5, AUC = 0.860).
